# Clinically used selective estrogen receptor modulators affect different steps of macrophage-specific reverse cholesterol transport

**DOI:** 10.1038/srep32105

**Published:** 2016-09-07

**Authors:** María E. Fernández-Suárez, Joan C. Escolà-Gil, Oscar Pastor, Alberto Dávalos, Francisco Blanco-Vaca, Miguel A. Lasunción, Javier Martínez-Botas, Diego Gómez-Coronado

**Affiliations:** 1 Servicio de Bioquímica-Investigación, Hospital Universitario Ramón y Cajal, IRYCIS, Madrid, Spain; 2 CIBER de Fisiopatología de la Obesidad y Nutrición (CIBEROBN), Madrid, Spain; 3 Institut d’Investigacions Biomèdiques (IIB) Sant Pau, Barcelona, Spain; 4 Departament de Bioquímica i Biología Molecular, Universitat Autònoma de Barcelona, Bellaterra, Spain; 5 CIBER de Diabetes y Enfermedades Metabólicas Asociadas (CIBERDEM), Spain; 6 Servicio de Bioquímica Clínica, Hospital Universitario Ramón y Cajal, IRYCIS, Madrid, Spain; 7 Instituto Madrileño de Estudios Avanzados (IMDEA)-Alimentación, Madrid, Spain

## Abstract

Selective estrogen receptor modulators (SERMs) are widely prescribed drugs that alter cellular and whole-body cholesterol homeostasis. Here we evaluate the effect of SERMs on the macrophage-specific reverse cholesterol transport (M-RCT) pathway, which is mediated by HDL. Treatment of human and mouse macrophages with tamoxifen, raloxifene or toremifene induced the accumulation of cytoplasmic vesicles of acetyl-LDL-derived free cholesterol. The SERMs impaired cholesterol efflux to apolipoprotein A-I and HDL, and lowered ABCA1 and ABCG1 expression. These effects were not altered by the antiestrogen ICI 182,780 nor were they reproduced by 17β-estradiol. The treatment of mice with tamoxifen or raloxifene accelerated HDL-cholesteryl ester catabolism, thereby reducing HDL-cholesterol concentrations in serum. When [^3^H]cholesterol-loaded macrophages were injected into mice intraperitoneally, tamoxifen, but not raloxifene, decreased the [^3^H]cholesterol levels in serum, liver and feces. Both SERMs downregulated liver ABCG5 and ABCG8 protein expression, but tamoxifen reduced the capacity of HDL and plasma to promote macrophage cholesterol efflux to a greater extent than raloxifene. We conclude that SERMs interfere with intracellular cholesterol trafficking and efflux from macrophages. Tamoxifen, but not raloxifene, impair M-RCT *in vivo*. This effect is primarily attributable to the tamoxifen-mediated reduction of the capacity of HDL to promote cholesterol mobilization from macrophages.

Reverse cholesterol transport (RCT) is the process by which high-density lipoproteins (HDL) convey cholesterol from peripheral cells to the liver for excretion into bile and feces^1^. Promotion of RCT from cholesterol-laden macrophages (M-RCT) present in the arterial wall is considered a major anti-atherogenic function of HDL^2^,^3^. The removal of excessive cholesterol from cells by HDL or lipid-poor apolipoprotein (apo) A-I constitutes the first step in RCT^1^. Cholesterol efflux is mainly mediated by ATP-binding cassette transporter (ABC) A1 and ABCG1, with lipid-poor apoA-I and mature HDL particles, respectively, serving as acceptors. Additionally, scavenger receptor class B type I (SR-BI) can also promote cholesterol export to mature HDL. In the liver, SR-BI mediates the selective uptake of HDL cholesterol. Subsequently, cholesterol can be converted to bile acids or be directly excreted to the bile through the heterodimeric transporter formed by ABCG5 and ABCG8 (ABCG5/G8). These four ABC proteins are transcriptionally upregulated by the liver X receptor (LXR), which is activated by oxysterols generated in response to increased cellular cholesterol content^4^. Macrophages can take up chemically modified low-density lipoproteins (LDL), such as oxidized LDL and acetylated LDL (AcLDL), a model of modified LDL used *in vitro*, through scavenger receptors, which are not regulated by cellular cholesterol content. This leads to the formation of cholesterol-laden macrophages, similar to those present in atherosclerotic lesions, by deposition of cholesteryl ester droplets^5^. Intracellular cholesterol trafficking plays a key role in cholesterol homeostasis^6^. Receptor-bound lipoproteins are transported to late endosomes/lysosomes (LE/L) where cholesteryl esters are hydrolyzed to free cholesterol. The subsequent egress of cholesterol from this compartment makes it available to other intracellular organelles, thus allowing cholesterol re-esterification by acyl-coenzyme A:cholesterol acyltransferase (ACAT) and the generation of LXR-activating oxysterols. Free cholesterol released from LE/L and from rehydrolyzed cholesteryl ester droplets can also move to the plasma membrane and thus be available for efflux out of the cell. Moreover, cholesterol can inhibit cholesterol biosynthesis and LDL receptor expression by suppressing the sterol regulatory element-binding protein (SREBP)-2 pathway^7^.

Selective estrogen receptor modulators (SERMs) are non-steroidal molecules that bind to estrogen receptors (ERs) and which are widely prescribed for the treatment and prevention of breast cancer, osteoporosis and ovulatory dysfunction^8^. SERMs display an estrogen-agonist or estrogen-antagonist effect depending on the tissue targeted, although different ER-independent effects have also been reported^9^,^10^,^11^. Tamoxifen (TAM) and toremifene (TOR), which are triphenylethylene derivatives differing only by the presence of a chlorine atom in the ethyl chain of TOR, are used to treat breast cancer. Raloxifene (RAL) is a benzothiophene derivative indicated for the treatment and prevention of osteoporosis in postmenopausal women^8^. In addition to their therapeutic effects, SERMs cause profound alterations in cellular and whole-body cholesterol homeostasis. These drugs consistently decrease total and LDL-cholesterol concentrations^12^, an effect that may be mediated by both the inhibition of cholesterol biosynthesis^13^,^14^,^15^ and the increase of LDL receptor activity^11^,^15^,^16^. Moreover, SERMs inhibit ACAT-mediated cholesterol esterification^11^,^17^. However, whether or not SERMs are cardioprotective in humans is subject to debate^8^,^18^,^19^, although it has been reported in different mouse models that TAM reduces atherosclerosis concurrent with a decrease of HDL-cholesterol^20^,^21^,^22^. Such an atheroprotective effect in mouse has been linked to the upregulation of transforming growth factor-β in the artery wall^20^,^21^,^22^. However, the effects of SERMs on the RCT pathway are unknown.

In human cell lines and primary lymphocytes we have demonstrated that TAM, RAL and TOR increase LDL receptor expression by impairing the egress of LDL-derived cholesterol from LE/L, thus blocking the transport of cholesterol to the endoplasmic reticulum and the subsequent inhibition of the SREBP-2 pathway^11^,^15^. These effects were independent of ERs^11^. We hypothesized that the SERM-mediated interference with cholesterol trafficking in macrophages may limit cholesterol efflux from these cells, thus hampering the macrophage-to-feces cholesterol transport *in vivo*. Therefore, we assessed the effect of clinically used SERMs on macrophage cholesterol trafficking and efflux and on the whole M-RCT pathway *in vivo*.

## Results

### SERMs impaired intracellular AcLDL-derived cholesterol trafficking and cholesterol efflux in macrophages

**Figure 1:**
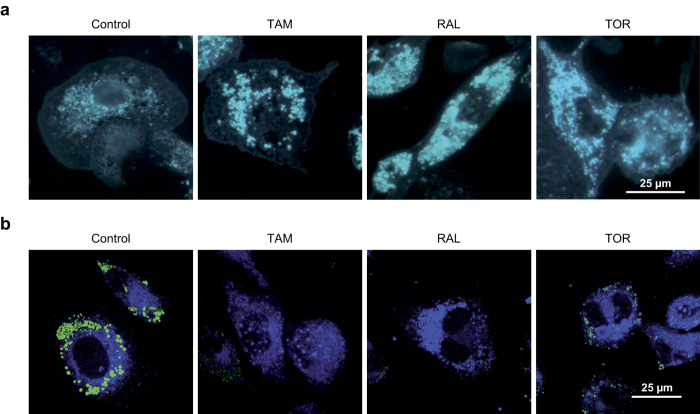
Effect of SERMs on free cholesterol and nonpolar lipid distribution in THP-1 macrophages. Cells were treated with AcLDL and vehicle (Control) or 10 μM tamoxifen (TAM), raloxifene (RAL) or toremifene (TOR). (**a**) Fluorescence microscopy of stainings with filipin for free cholesterol. (**b**) Confocal microscopy of costainings with filipin (blue) and Bodipy (green) for nonpolar lipids. Photographs are representative examples from three independent experiments.

We first determined the effect of TAM, RAL and TOR on the cholesterol content of THP-1 macrophages exposed to AcLDL for 24 h. AcLDL increased total, free and esterified cholesterol content, but these were not altered by treatment with any of the SERMs (Supplementary Fig. S1a). The distribution of free cholesterol in THP-1 macrophages was analyzed by addition of 1,1′-dioctadecyl-3,3,3,3′-tetramethylindocarbocyanineperchlorate (DiI)-labeled AcLDL and filipin staining. As shown in Fig. 1a, in the control condition filipin-positive structures displayed a diffuse distribution in the cytoplasm, with distinguishable small vesicles. The addition of the SERMs caused the appearance of abundant and larger cytoplasmic, filipin-positive vesicles, indicative of the accumulation of free cholesterol. These vesicles co-localized with DiI, suggesting that the accumulated cytoplasmic free cholesterol derives from internalized AcLDL and is retained in LE/L (Supplementary Fig. S2). When nonpolar lipids were stained with Bodipy and analyzed by confocal microscopy, it was observed that SERM treatment decreased the number of Bodipy-positive droplets (Fig. 1b). Given this effect on nonpolar lipids in the absence of changes in esterified cholesterol mass, triacylglycerols were measured. AcLDL increased triacylglycerol mass relative to lipoprotein-deficient serum (LPDS) alone, whereas any of the SERMs abrogated such an increase (Supplementary Fig. S1c). Therefore, the reduction of nonpolar lipids after a 24-h treatment with SERMs correlates with the decrease of the mass of triacylglycerols, but not with that of esterified cholesterol.

The enzyme responsible for re-esterification of lipoprotein-derived cholesterol is ACAT and resides in the endoplasmic reticulum. When cholesterol exits the lysosomes, the cellular cholesterol pool expands and ACAT is activated. Thus, the effect of SERMs on ACAT activity was determined. As shown in Supplementary Fig. S1d, the three SERMs, but more intensely RAL and TOR, effectively opposed the AcLDL-mediated stimulation of cholesterol esterification. This contrasts with the lack of effect of SERMs on esterified cholesterol mass. We, then, hypothesized that 24 h of SERM treatment is not sufficient to alter the cellular content of esterified cholesterol, nor that of free cholesterol. To test this hypothesis, the treatment time was prolonged to 48 h (Supplementary Fig. S1b). While LPDS treatment markedly reduced free and esterified cholesterol mass, macrophages loaded with AcLDL showed increased total cholesterol mass as compared with those loaded for 24 h, which was accounted by an elevation of the esterified cholesterol mass (compare Supplementary Fig. S1a,b). SERMs opposed this increase of esterified cholesterol, RAL and TOR, the most active at inhibiting ACAT activity, being especially effective. Simultaneously, SERMs tended to increase the free cholesterol content as compared with the treatment with AcLDL alone (control), the effect of RAL being statistically significant (Supplementary Fig. S1b). As a consequence of the opposite effects of SERMs on free and esterified cholesterol, the mass of total cholesterol remained similar to that of control cells.

**Figure 2:**
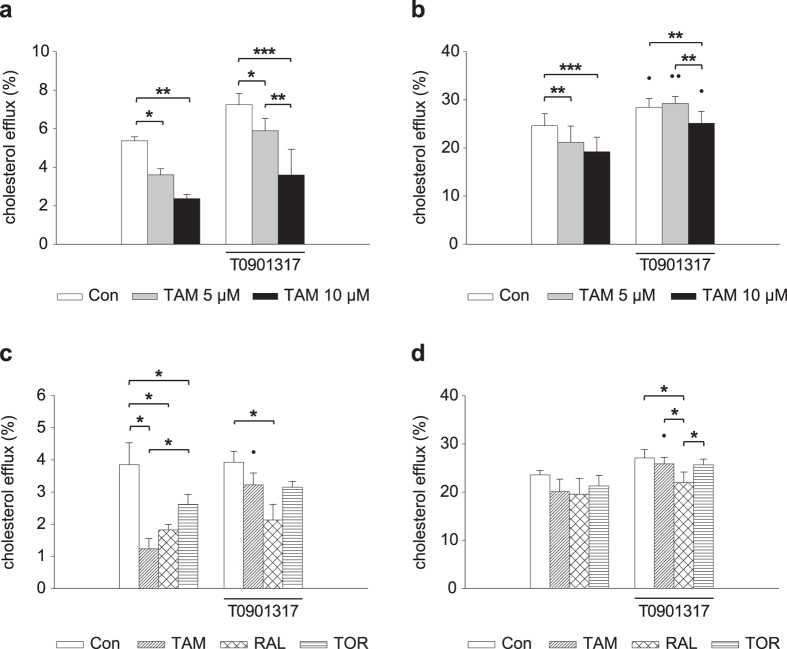
Effect of SERMs on AcLDL-derived cholesterol efflux from THP-1 macrophages. (**a**,**b**) Cells were treated with [^3^H]cholesterol-AcLDL and vehicle (Con, control) or the indicated concentrations of tamoxifen (TAM) and in the absence or presence of T0901317 (1 μM). The cells were washed and cholesterol efflux was measured in the presence of apoA-I (**a**) or HDL (**b**) at 8 h. (**c,d**) Cells were treated with [^3^H]cholesterol-AcLDL and vehicle (Con, control) or tamoxifen (TAM), raloxifene (RAL) or toremifene (TOR) (10 μM) and in the absence or presence of T0901317 (1 μM). Then, cells were washed and cholesterol efflux was measured in the presence of apoA-I (**c**) or HDL (**d**) at 8 h. Data are mean ± SEM of 3 or 4 independent experiments. *P < 0.05, **P < 0.01, ***P < 0.001; ^•^*P* < 0.05, ^••^*P* < 0.01 between conditions only differing in the presence of T0901317.

Next we analyzed the effect of the SERMs on AcLDL-derived cholesterol efflux from THP-1 macrophages, for which cells were loaded with [^3^H]cholesterol-labeled AcLDL in the presence of the SERMs. This treatment did not affect macrophage labeling with [^3^H]cholesterol-AcLDL (Supplementary Fig. S3). First, we tested the effect of different concentrations of TAM. This drug decreased apoA-I-mediated cholesterol export in a dose-dependent manner (Fig. 2a). HDL-mediated cholesterol efflux was also reduced (Fig. 2b), although to a lower extent than in the presence of apoA-I. It has been reported that the LXR agonist T0901317 stimulates the trafficking from LE/L to the plasma membrane and subsequent efflux of AcLDL-derived cholesterol^23^. Therefore, we tested the effect of T0901317 on TAM-mediated decrease of cholesterol efflux from THP-1 macrophages. T0901317 modestly increased cholesterol efflux to both apoA-I and HDL, but the reduction by TAM persisted (Fig. 2a,b). Then, the effect of RAL and TOR at a 10 μM concentration was also examined. Like TAM, those SERMs lowered cholesterol efflux to apo-A-I (Fig. 2c) and, more weakly, to HDL (Fig. 2d), and these effects essentially persisted in the presence of T0901317. As a whole, these results suggest that the SERMs interfere with AcLDL-derived cholesterol intracellular trafficking and subsequent export from macrophages.

**Figure 3:**
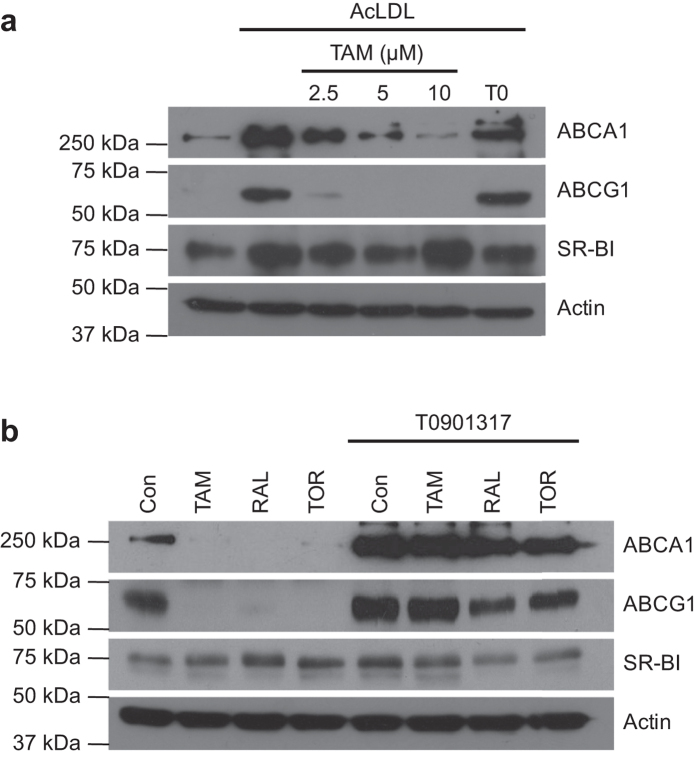
Effect of SERMs on ABCA1, ABCG1 and SR-BI protein expression in THP-1 macrophages. (**a**) Cells were incubated in the absence or presence of AcLDL and the indicated concentrations of tamoxifen (TAM) or T0901317 (T0, 1 μM). (**b**) Cells were treated with AcLDL and vehicle (Con, control) or tamoxifen (TAM), raloxifene (RAL) or toremifene (TOR) (10 μM) and in the absence or presence of T0901317 (1 μM).

To get a deeper insight into the mechanism underlying the decrease of cholesterol efflux by SERMs, the expression of ABCA1, ABCG1 and SR-BI was determined. As expected, AcLDL markedly increased the protein levels of ABCA1 and ABCG1. TAM dose-dependently opposed the effects of AcLDL (Fig. 3a). These effects paralleled those on the respective mRNA levels (Supplementary Fig. S4). RAL and TOR, tested at 10 μM, also decreased ABCA1 and ABCG1 protein levels (Fig. 3b). In contrast, the three SERMs slightly increased SR-BI expression (Fig. 3a,b). On the other hand, the T0901317-induced stimulation of ABCA1 and ABCG1 expression was only partially prevented by the SERMs (Fig. 3b).

The SERM-mediated suppression of ABCA1 and ABCG1 expression may contribute, by itself, to the lowering of AcLDL-derived cholesterol efflux. To test this possibility, THP-1 macrophages were labeled with [^3^H]cholesterol added in ethanol, which preferentially labels cellular pools other than LE/L^24^. Subsequently, cells were treated with AcLDL plus TAM or vehicle and, finally, cholesterol efflux was measured as above. By this procedure, the effect of TAM on [^3^H]cholesterol efflux is independent of its effect on intracellular trafficking of AcLDL-derived cholesterol. TAM dose-dependently inhibited both apoA-I-mediated and, less effectively, HDL-mediated cholesterol efflux (Supplementary Fig. S5). This suggests that SERMs decrease cholesterol export from THP-1 macrophages not only by lowering the availability of lipoprotein cholesterol, but also by impairing the AcLDL-mediated induction of ABCA1 and ABCG1.

To confirm the effects of SERMs on human primary macrophages, human monocyte-derived macrophages were treated as described above. The three SERMs also caused the accumulation of cytoplasmic free cholesterol and the reduction of nonpolar lipids (Supplementary Fig. S6). Simultaneously, the SERMs suppressed the expression of ABCA1 and ABCG1, an effect that was abrogated by T0901317, whereas they increased SR-BI expression independently of the presence of T0901317 (Supplementary Fig. S7). The expression of CD36 and NPC1 was not appreciably altered (Supplementary Fig. S7). Preliminary studies showed that apoA-I- and HDL-mediated cholesterol efflux from [^3^H]cholesterol-AcLDL-labeled primary macrophages was less efficient than from THP-1 macrophages (results not shown), for which reason the efflux phase was prolonged up to 24 h, throughout which the SERMs were kept in the media. Again, the SERMs decreased AcLDL-derived cholesterol efflux to apoA-I and, to a lower extent, HDL, which was at least partially prevented by the addition of T0901317 (Supplementary Fig. S8).

**Figure 4:**
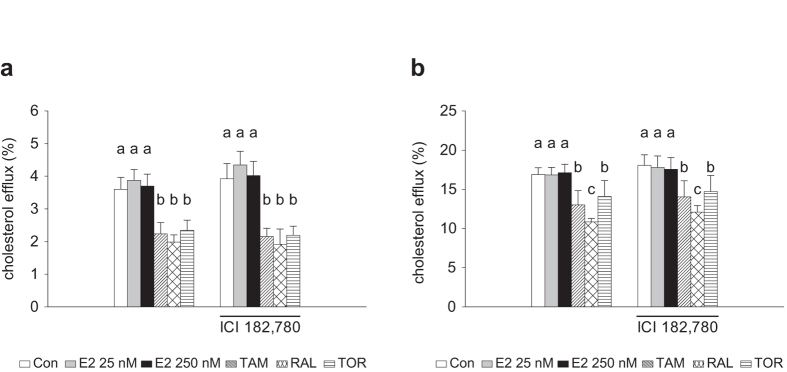
Effect of SERMs, ICI 182,780 and 17β-estradiol on cholesterol efflux from mouse peritoneal macrophages. Cells were labelled with [^3^H]cholesterol added in ethanol and then treated with AcLDL and vehicle (Con, control) or tamoxifen (TAM), raloxifene (RAL) or toremifene (TOR) (10 μM), or the indicated concentrations of 17β-estradiol (E2) and in the absence or presence of ICI 182,780 (1 μM). Subsequently cholesterol efflux was measured in the absence or presence of apoA-I (**a**) or HDL (**b**) at 8 h. Data are mean ± SEM of macrophages from 4 or 5 mice. Bars with different letters are statistically different (*P* < 0.05).

We evaluated whether ERs were involved in the effects of the SERMs. The expression of both *ESR1* and *ESR2* in THP-1 macrophages was undetectable (Ct >39 cycles) and SERM treatment did not change this expression, suggesting that the effects of SERMs were independent of ERs. This issue was further explored in mouse peritoneal macrophages. As shown in Supplementary Fig. S9, when these cells were treated with the SERMs, as indicated for human macrophages, all three drugs induced the accumulation of free cholesterol-rich vesicles and markedly reduced the number of nonpolar lipid droplets. On the other hand, the SERMs inhibited cholesterol efflux from mouse macrophages to both apoA-I and HDL (Fig. 4). The simultaneous addition of ICI 182,780, a selective ER down-regulator, did not alter the effect of any SERM on cytoplasmic free cholesterol and nonpolar lipid accumulation (Supplementary Fig. S9) or on cholesterol efflux (Fig. 4). Consistently, 17β-estradiol, the natural ligand of ERs, was unable to influence intracellular cholesterol distribution (Supplementary Fig. S9) and cholesterol efflux to apoA-I or HDL (Fig. 4) when compared with untreated macrophages.

### TAM and RAL decreased HDL-cholesterol concentrations, but only TAM reduced macrophage-specific RCT

**Figure 5:**
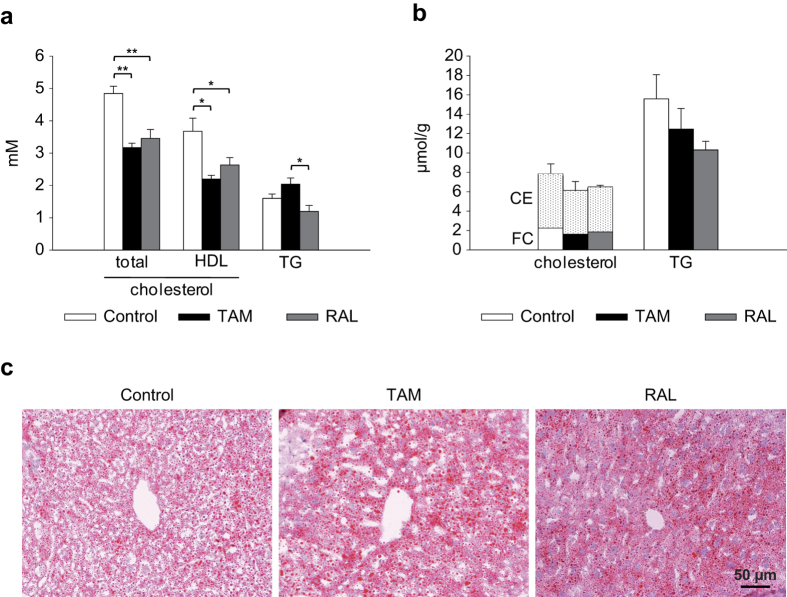
Effect of tamoxifen and raloxifene on serum and hepatic lipid concentrations in mice. Mice were fed a western-type diet for 4 weeks and were treated with tamoxifen (TAM), raloxifene (RAL) or vehicle (Control) for the last 10 days. (**a**) Cholesterol, HDL-cholesterol and triacylglycerol (TG) serum concentrations. (**b**) Hepatic free cholesterol (FC), cholesteryl ester (CE) and triacylglycerol concentrations. Data are mean ± SEM of 5 mice per group. *P < 0.05, **P < 0.01. (**c**) Histological stainings with Oil Red O for nonpolar lipids and hematoxylin in liver sections.

Next we assessed the impact of SERMs on M-RCT in mice. The effects of TAM, as a representative of the two triphenylethylene derivatives used herein, and RAL were studied. Mouse peritoneal macrophages were radiolabeled with [^3^H]cholesterol, loaded with AcLDL and treated with TAM, RAL or vehicle as described above. Mice given a Western-type diet were treated with TAM, RAL or vehicle by oral gavage for 10 days. Forty-eight hours before sacrifice, they received an intraperitoneal injection of radiolabeled macrophages previously treated *ex vivo* with the same SERM or vehicle, respectively. Both TAM and RAL reduced serum and HDL-cholesterol levels as compared with control mice, whereas TAM increased serum triacylglycerol concentrations relative to RAL (Fig. 5a). There were no significant differences in hepatic cholesterol and triacylglycerol contents between the treatments (Fig. 5b). Consistently, Oil Red O staining of liver sections showed abundant neutral lipid droplets with all the treatments (Fig. 5c). However, filipin staining did not result in sufficiently well-resolved images to enable the distribution of free cholesterol in the hepatocytes to be discerned (Supplementary Fig. S10).

**Figure 6:**
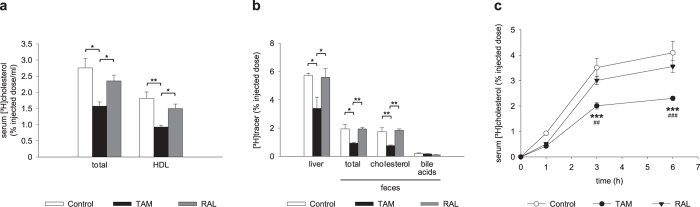
Effects of tamoxifen and raloxifene on macrophage-specific RCT. Mice were fed a western-type diet for 4 weeks and treated with tamoxifen (TAM), raloxifene (RAL) or vehicle (Control) for the last 10 days. Forty-eight hours before sacrifice, mice were injected intraperitoneally with [^3^H]cholesterol-labelled, AcLDL-loaded mouse peritoneal macrophages that had been treated *ex vivo* with the same drug or vehicle, respectively. (**a**) Serum total and HDL-associated [^3^H]cholesterol at 48 h. (**b**) Liver [^3^H]cholesterol at 48 h and excretion of total [^3^H]tracer, [^3^H]cholesterol and [^3^H]bile acids in feces over 48 h. Data are mean ± SEM of 5 mice per group. *P < 0.05, **P < 0.01. (**c**) Serum [^3^H]cholesterol at early time points. Data are mean ± SEM of 5 mice per group. ***P < 0.001 versus Control; ^##^*P* < 0.01, ^###^*P* < 0.001 versus RAL.

TAM treatment reduced serum [^3^H]tracer radioactivity at 48 h after macrophage injection, which was mostly attributable to reduced [^3^H]tracer in HDL (Fig. 6a). RAL, on the other hand, produced no significant changes versus control mice. Similarly, TAM, but not RAL, lowered hepatic [^3^H]tracer values (Fig. 6b). Fecal [^3^H]tracer excretion over 48 h was also reduced by TAM, RAL again producing no effect (Fig. 6b). Therefore, although both TAM and RAL decreased concentrations of HDL-cholesterol, only TAM impaired M-RCT. The lower recovery of [^3^H]tracer in serum, liver and feces of TAM-treated mice suggested a lower [^3^H]cholesterol mobilization from macrophages. To test this possibility, the amount of [^3^H]cholesterol in serum was measured at early time points after injection of labeled macrophages. The rate of appearance of [^3^H]cholesterol in serum was reduced by TAM and, to a much lower extent, RAL treatment (Fig. 6c). TAM significantly decreased serum [^3^H]cholesterol at 3 and 6 h versus vehicle and RAL, whereas RAL did not cause a significant effect at any time point.

### TAM and RAL altered HDL composition, cholesterol efflux capacity and catabolism to different degrees

**Figure 7:**
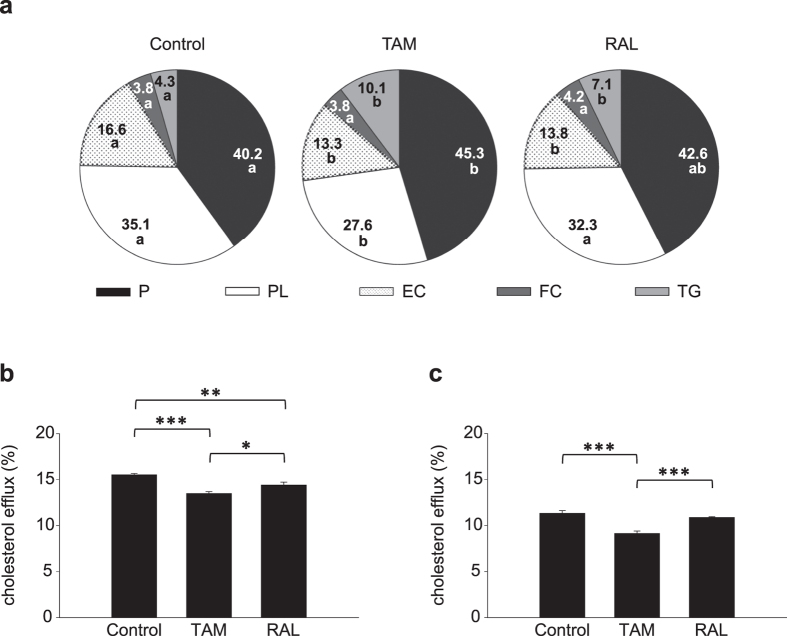
Effect of tamoxifen and raloxifene on HDL composition and *ex vivo* cholesterol efflux capacity. Plasma HDL was isolated from mice fed a western-type diet for 4 weeks and treated with tamoxifen (TAM), raloxifene (RAL) or vehicle (Control) for the last 10 days. (**a**) Pie chart of the chemical composition of HDL. Values are mean percentages of 4 mice per group. Sectors of a given component not sharing a letter are statistically different (*P* < 0.05). P, protein; PL, phospholipids; EC, esterified cholesterol; FC, free cholesterol; TG, triacylglycerols. (**b,c**) [^3^H]Cholesterol-labelled and AcLDL-loaded mouse peritoneal macrophages were incubated in the absence or presence of HDL (40 μg/mL) (**b**) or whole plasma (1%) (**c**) for 8 h to measure cholesterol efflux. *P < 0.05, **P < 0.01, ***P < 0.001.

We characterized HDL composition and *ex vivo* cholesterol efflux capacity, for which plasma HDL was isolated from mice treated as indicated above. As shown in Fig. 7a, TAM decreased the percentage of phospholipids and esterified cholesterol and increased those of triacylglycerols and total protein, whereas RAL only changed the content of neutral lipids. HDL from SERM-treated animals was less effective in promoting cholesterol efflux from mouse peritoneal macrophages than that from vehicle-treated animals, TAM displaying a slightly but significantly greater effect than RAL (Fig. 7b). When the efflux to whole plasma was examined, it was found that TAM, but not RAL, reduced the capacity of plasma to promote cholesterol efflux (Fig. 7c).

**Figure 8:**
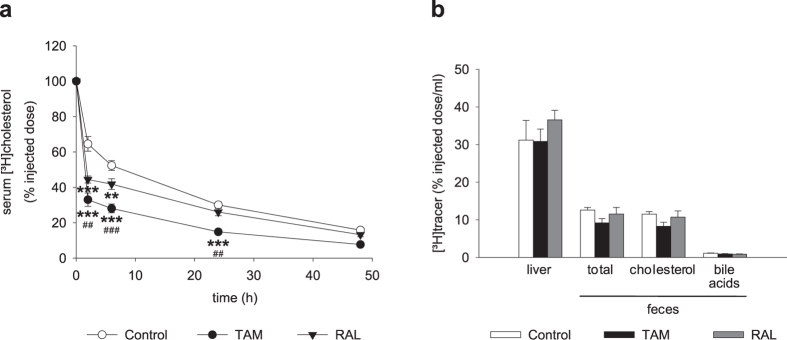
Effect of tamoxifen and raloxifene on the clearance of [^3^H]cholesteryl oleate-labelled HDL in mice. Plasma HDL was isolated from mice fed a western-type diet for 4 weeks and treated with tamoxifen (TAM), raloxifene (RAL) or vehicle (Control) for the last 10 days. HDL were labelled with [^3^H]cholesteryl oleate and injected intravenously into mice at day 8 of treatment with the same SERM or vehicle as the HDL donor mice. (**a**) Decay curve of [^3^H]cholesteryl oleate-labelled HDL in serum. (**b**) Liver [^3^H]cholesterol at 48 h and excretion of total [^3^H]tracer, [^3^H]cholesterol and [^3^H]bile acids in feces over 48 h. Data are mean ± SEM of 4 mice per group. **P < 0.01, ***P < 0.001 versus Control; ^##^*P* < 0.01, ^###^*P* < 0.001 versus RAL.

To assess the effect of these SERMs on the catabolism and fate of cholesterol from the HDL core, each mouse was injected intravenously with HDL isolated from donor mice of the same treatment group and labeled with [^3^H]cholesteryl oleate. The SERMs accelerated the clearance of HDL-[^3^H]cholesteryl oleate from serum, TAM having the highest effect (Fig. 8a). There were no differences between the treatments in the amount of hepatic [^3^H]tracer (Fig. 8b). Moreover, fecal excretion of HDL-derived [^3^H]cholesterol showed a downward, non-significant trend in TAM-treated mice (Fig. 8b).

### TAM and RAL produced similar effects on the expression of hepatic cholesterol transporters

**Figure 9:**
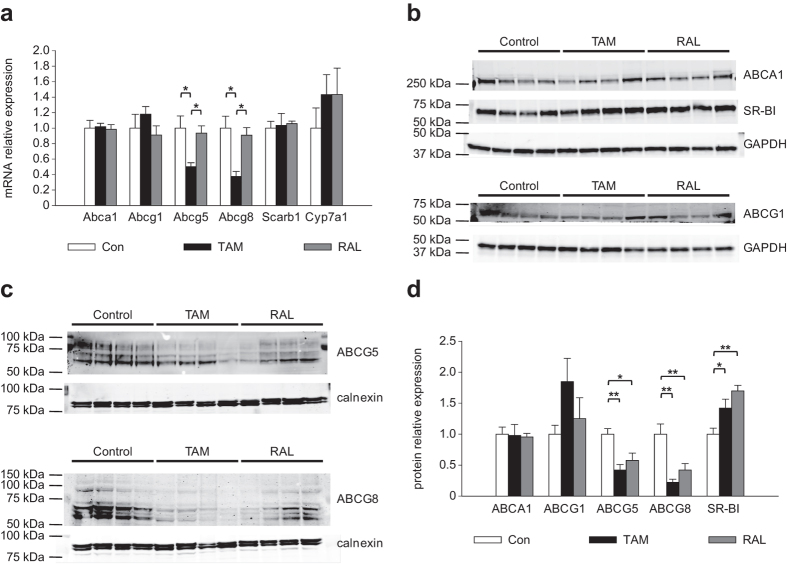
Effect of tamoxifen and raloxifene on the hepatic expression of cholesterol transporters. Mice were fed a western-type diet for 4 weeks and were treated with tamoxifen (TAM), raloxifene (RAL) or vehicle (Control, Con) for the last 10 days. (**a**) Gene expression analysis by real-time PCR. Data are mean ± SEM of 5 mice per group and are expressed as the relative amount of mRNA compared to the level in the control condition. (**b**,**c**) Western blot analysis of liver (**b**) and liver membrane fraction (**c**) lysates. (**d**) Scan densitometry quantification of protein levels of the blottings shown in (**b**,**c)**. Data are mean ± SEM of the ratios between the levels of each protein and those of GAPDH or calnexin, respectively. *P < 0.05, **P < 0.01.

Next, we determined the expression of genes relevant for cholesterol transport and its conversion to bile acids in the liver. As shown in Fig. 9a, RAL did not alter the content of the mRNA for any of the genes studied, whereas TAM decreased those of *Abcg5* and *Abcg8*. To ascertain whether these effects translated into changes in protein expression, western blot assays were performed. Neither TAM nor RAL altered the expression of ABCA1 or ABCG1, but both SERMs increased SR-BI expression (Fig. 9b,d). ABCG5 and ABCG8 were analyzed in liver membrane fractions. Several bands appeared for both proteins (Fig. 9c), which have been reported to correspond to different glycosylated forms ranging from approximately 65 to 100 kDa^25^,^26^,^27^. Both TAM and RAL decreased the expression of the different forms of ABCG5 and ABCG8 (Fig. 9c,d).

## Discussion

In this study we provide evidence that SERMs impair cholesterol efflux from macrophages *in vitro* and that TAM, but not RAL, reduces M-RCT *in vivo*. SERMs had the ability to alter several steps of RCT, since they decreased cholesterol mobilization from macrophages, reduced the capacity of HDL to promote cholesterol efflux, accelerated plasma HDL catabolism and lowered hepatic expression of ABCG5/G8. These effects, many of which were common to TAM and RAL, were generally more pronounced in TAM-treated mice and seem to have a differential impact on M-RCT.

TAM, RAL and TOR inhibited cholesterol efflux from human and mice macrophages *in vitro* in association with an inhibition of the egress of AcLDL-derived cholesterol from LE/L. This trafficking blockade is in full agreement with the effect of SERMs on native LDL-derived cholesterol in other cell types^11^,^15^. As a consequence, lipoprotein-derived cholesterol is unavailable for efflux from the cell and, consistent with previous findings^11^,^17^, ACAT-mediated cholesterol esterification. These effects were observed at concentrations of TAM and TOR within the range of those achieved in the plasma of treated patients^28^,^29^, although higher than those achieved with RAL, which has poor bioavailability^28^. We also showed that ERs were not involved in the inhibition of macrophage cholesterol efflux by SERMs, which agrees with our previous studies demonstrating that the SERM-mediated stimulation of the SREBP pathway and LDL uptake is independent of ERs^11^,^15^. On the other hand, SERMs suppressed the lipoprotein cholesterol-induced expression of ABCA1 and ABCG1, suggesting an impaired signalling through LXR, a key transcriptional regulator of such transporters. This probably results from a failure to generate cholesterol-derived oxysterols required to activate LXR, similar to what occurs in Niemann–Pick disease type C fibroblasts^30^, which are unable to export cholesterol from lysosomes and, consequently, have deficient upregulation of ABCA1 and cholesterol efflux to apoA-I^32^. The inhibition of the AcLDL-induced increase of triacylglycerol mass by SERMs is also consistent with an impairment of LXR activation, given that LXR is an established lipogenic factor^32^. The reason for the higher inhibition of cholesterol efflux to apoA-I than to HDL, as found herein, may be due to a compensating effect on the export to HDL exerted by the SERM-mediated increased SR-BI expression, although the mechanism whereby SERMs affect SR-BI is unknown.

The results presented here are in line with the reduction of cholesterol efflux from ABCA1- and ABCG1-deficient macrophages^33^. It has been reported that the deficiency of these transporters impairs M-RCT in mice and that they have an additive effect^33^,^34^. This prompted us to study the effect of TAM and RAL on M-RCT. Surprisingly, only TAM inhibited M-RCT, suggesting that factors other than macrophage cholesterol efflux determine the differential effect of TAM and RAL on M-RCT. However, both drugs reduced serum HDL-cholesterol concentrations, which supports the concept that the rate of M-RCT is not necessarily a reflection of HDL-cholesterol concentrations, as previously found by others^2^,^3^. The decrease of HDL-cholesterol by SERMs is coincident with an accelerated clearance of HDL-cholesteryl esters and increased hepatic SR-BI expression, a key determinant of HDL-cholesterol uptake^35^. Enhanced SR-BI protein expression, but unchanged mRNA levels, and reduced HDL-cholesterol has also been found in rats treated with the SERM acolbifene^36^. Hepatic ABCA1 expression, which is essential for HDL biogenesis^37^, was unaltered. Therefore, the likely mechanism for the HDL-cholesterol decrease caused by SERMs is increased catabolism.

In principle, an increased SR-BI-mediated HDL cholesterol uptake by the liver would favor the fecal excretion of macrophage-derived cholesterol^38^, but this was not observed with any drug. Interestingly, both TAM and RAL inhibited the expression of hepatic ABCG5 and ABCG8 protein, which form a heterodimer that plays a key role in biliary cholesterol excretion^39^,^40^. The fact that both SERMs decreased the expression of hepatic ABCG5/G8 suggests that this transporter does not have a major role in the effect of TAM on M-RCT. The moderate effect of TAM on fecal [^3^H]cholesterol excretion after intravenously injecting HDL-[^3^H]cholesteryl oleate also supports this conclusion. Alternative mechanisms of cholesterol excretion may have compensated for the low expression of ABCG5/G8. Previous reports have shown that SR-BI can mediate biliary cholesterol secretion and M-RCT independently of ABCG5/G8 in mice^41^,^42^. Moreover, an intestinal route for cholesterol excretion and M-RCT has been described^43^,^44^. These mechanisms may have also contributed to prevent hepatic cholesterol accumulation in SERM-treated mice despite increased SR-BI and decreased ABCG5/G8 expression.

The lower fecal excretion of macrophage-derived cholesterol with TAM treatment was associated with reductions in the rate of appearance of [^3^H]cholesterol in serum and its recovery in liver at 48 h. In contrast, RAL only marginally decreased the rate of appearance of [^3^H]cholesterol in serum and had no effect on its recovery in liver and feces. However, when the transport of [^3^H]cholesterol to feces was measured after the intravenous injection of HDL-[^3^H]cholesteryl oleate, which introduces the [^3^H]tracer downstream of macrophage efflux, neither TAM nor RAL treatment significantly altered fecal [^3^H]tracer excretion. Collectively these results suggest that TAM and RAL have a differential impact on the capacity to mobilize cholesterol from macrophages and, thereby, its subsequent transport to feces. Additionally, we found that HDL from SERM-treated mice had a reduced capacity to promote macrophage cholesterol efflux *in vitro*, TAM being more active than RAL. Moreover, the efflux capacity of plasma was lowered by TAM, but not RAL treatment. Therefore, an impaired activity of HDL as acceptor of macrophage cholesterol may contribute to the lower mobilization and macrophage-to-feces transport of cholesterol in TAM-treated mice. In line with this, two reports have indicated that the activity of HDL to promote macrophage cholesterol efflux plays a principal role in the ability of T0901317 to stimulate M-RCT^45^,^46^. The lower efflux potential of HDL and plasma from TAM-treated mice may be due to the lower phospholipid content of such particles, whose phospholipid/protein ratio decreased by 30% and 20% versus HDL from, respectively, vehicle- and RAL-treated mice. It has been proposed that phospholipids are the HDL component that best reflects the serum’s efflux efficiency^47^,^48^,^49^. The lower phospholipid content may also contribute to the accelerated clearance of HDL *in vivo*, because HDL modification by phospholipases makes HDL cholesteryl esters more susceptible to SR-BI-mediated selective uptake^50^.

As mentioned above, the effects of SERMs on intracellular trafficking and efflux of cholesterol are independent of ERs. The involvement of ERs in at least some of the effects of SERMs *in vivo* cannot be ruled out. However, the effects produced by estrogen treatment on the hepatic expression of cholesterol transporters in mice^51^,^52^ and rats^53^,^54^ differ from those observed herein. ABCG5 and ABCG8, in particular, do not appear to be regulated by estrogen^52^. These transporters are known LXR targets required for LXR agonist-mediated stimulation of cholesterol excretion^55^, the liver ABCG5/G8 heterodimer being a key determinant of the M-RCT stimulation induced by LXR agonists and a high-cholesterol diet^56^,^57^. By analogy with the effect of SERMs on cultured cells (reported here and elsewhere^11^,^15^), a disruption of intracellular cholesterol trafficking in hepatocytes may be involved in the reduction of ABCG5/G8 expression. However, we were unable to distinguish cytoplasmic deposition of free cholesterol in hepatocytes or alterations in free or esterified cholesterol liver content. Curiously, while both protein and mRNA levels of ABCG5 and ABCG8 were reduced at the end of the study in TAM-treated mice, only the protein levels were diminished by RAL. The reason for such a discrepancy between both SERMs is unknown. Perhaps it is due to a more potent transcriptional effect or higher bioavailability of TAM than RAL, thus increasing the likelihood of observing changes in mRNA over time. Actually, RAL has been found to have a lower bioavailability than TAM in humans^28^ and rats^58^,^59^. In a clinical setting, RAL is administered to patients at higher doses than TAM^28^, so the possibility that higher doses of RAL are able to diminish M-RCT cannot be ruled out.

Although ABCA1 and ABCG1 are also LXR targets, their hepatic expression was not affected by SERMs. This is consistent with the known differential regulation of these transporters and ABCG5/G8 by cholesterol in the liver. Hepatic ABCA1 expression is known to be regulated by a SREBP-2- and LXR-driven dual promoter system^60^. This may explain why hepatic ABCA1 mRNA is unaffected by feeding cholesterol to mice, whereas ABCG5/G8 mRNA levels are markedly induced^61^. Additionally, hepatic ABCA1 and ABCG1 mRNA expression is less sensitive to LXR agonists than their extrahepatic expression^62^,^63^. On the basis of these observations and our own data, we hypothesize that hepatic ABCA1 and ABCG1 expression shows a distinct response to SERM-mediated alteration of cholesterol homeostasis.

It should be noted that, although TAM treatment of mouse models of atherosclerosis consistently reduces HDL-cholesterol, it also abolishes lesion formation^20^,^21^,^22^. However, the cardioprotective effect of SERMs in humans is the subject of debate^8^,^18^,^19^. Moreover, SERMs do not usually lower HDL concentrations in humans, but consistently decrease LDL concentrations^12^, the predominant lipoprotein fraction in this species. Mice, in contrast to humans, lack the cholesteryl ester transfer protein. This transfers cholesteryl esters from HDL to apoB-containing lipoproteins which, through their subsequent liver uptake, contribute to RCT. In fact, it has been reported that the bulk of HDL cholesteryl esters is taken up by the liver after transfer to apoB-containing lipoproteins^64^. This fact may limit the impact of SERMs on HDL cholesterol catabolism and, hence, HDL concentrations. However, HDL is responsible for the net delivery of free cholesterol to the liver^64^. Given the differences in lipoprotein metabolism between humans and mice, the relevance of the present results for humans is uncertain. In any case, the possibility that the macrophage-to-feces cholesterol transport is altered in patients taking TAM, even despite unchanged HDL concentrations, cannot be discarded. On the other hand, recent findings enable us to suggest that the SERM-induced downregulation of ABCA1 and ABCG1 in macrophages may modulate inflammation^65^ and tumor immunity^66^,^67^.

In conclusion, the SERMs interfere with intracellular cholesterol trafficking and efflux from macrophages *in vitro*. However, TAM, but not RAL, impairs M-RCT *in vivo*. This effect is independent of the decrease of HDL levels caused by SERMs, but it is primarily attributable to the TAM-mediated reduction of the capacity of HDL to promote cholesterol mobilization from macrophages. Our data are suggestive of a negative effect of TAM on a major atheroprotective function of HDL that remains to be demonstrated in interventional studies with TAM-treated patients.

## Materials and Methods

### Animals

Wild-type C57BL/6 mice were fed a standard chow diet before the studies. All animal procedures were approved by the Institutional Animal Care Committee of the Institut de Recerca de l’Hospital de Sant Pau and Comité Ético de Experimentación Animal of the Hospital Universitario Ramón y Cajal and conducted in accordance with the approved guidelines.

### THP-1 cell culture and macrophage differentiation

THP-1 cells (ATCC TIB-202) were maintained in RPMI 1640 containing 10% heat-inactivated FBS, 2 mM glutamine, 100 U/mL penicillin, 100 U/mL streptomycin and 10 μg/mL gentamicin at 37 °C in a humidified atmosphere of 5% CO_2_. THP-1 monocytes (5 × 10^5^ cells/mL) were differentiated to macrophages with 50 ng/mL PMA for 48 h.

### Human monocyte-derived and mouse peritoneal macrophages

Human peripheral blood monocytes were isolated by density gradient centrifugation of buffy coats from male donors obtained from the Centro de Transfusiones de la Comunidad de Madrid and as described in Supplementary Methods. Peritoneal macrophages were isolated from male mice by lavage after intraperitoneal injection of thioglycolate as described in Supplementary Methods.

### Lipoproteins and LPDS

Human LDL was isolated, acetylated and labeled with the fluorescence probe 1,1′-dioctadecyl-3,3,3,3′-tetramethylindocarbocyanineperchlorate (DiI) as previously described^68^. Human HDL (1.063–1.21 kg/L) was isolated by sequential ultracentrifugation of fasting plasma. LPDS was prepared from fetal bovine serum (FBS) or charcoal/dextran-treated FBS (Gibco BRL, Life Technologies) by ultracentrifugation at a density of 1.21 kg/L.

### *In vitro* experimental design

Macrophages were pretreated with the corresponding medium supplemented with 10% LPDS for 24 h. Subsequently, TAM, RAL (Tocris Bioscience), TOR, E2 (Sigma-Aldrich), ICI 182,780 (Tocris Bioscience), T0901317 or vehicle (dimethyl sulfoxide, final concentration 0.044%) were added to the media as indicated, and 1 h later AcLDL-cholesterol (120 μg/mL) was added to all the treatments. Twenty four hours after the addition of the drugs, E2 or vehicle, the cells were washed and processed to perform the corresponding analysis. To assess the effects of ICI 182,780 and E2 in mouse peritoneal macrophages, DMEM without phenol red and supplemented with 10% charcoal/dextran-treated LPDS was used.

### Cholesterol efflux experiments

To analyze the efflux of AcLDL-derived cholesterol, [1,2-^3^H(N)]cholesterol-labeled AcLDL was prepared by incubating [1,2-^3^H(N)]cholesterol (Perkin-Elmer), previously dried on the tube wall, with AcLDL (1 μCi [1,2-^3^H(N)]cholesterol/120 μg AcLDL-cholesterol) overnight at room temperature. The different treatments of the macrophages (see above) were carried out in the presence of AcLDL-[^3^H]cholesterol (120 μg/mL). Then, the cells were exhaustively washed with PBS containing 0.1% BSA and equilibrated with RPMI 1640, 0.2% BSA for 1 h. On other occasions, macrophages were labeled with [1,2-^3^H(N)]cholesterol (0.5 μCi/mL), added in ethanol (final concentration 0.44%), in the medium supplemented with 10% LPDS for 24 h and, after being washed and equilibrated, they were treated with the drugs in the presence of AcLDL-cholesterol (120 μg/mL). Subsequently, the cells were incubated in RPMI 1640, 0.2% BSA supplemented or not with human apoA-I (Calbiochem) or HDL (50 μg/mL) for the indicated times. Then, the medium was collected and centrifuged at 10,000 × g for 10 min and the cells were lysed with 0.1 M NaOH, 0.1% sodium dodecyl sulfate. The radioactivity was determined in the medium and cell lysates. Cholesterol efflux was expressed as the percentage of the radioactivity released from the cells into the medium relative to the sum of radioactivity in cells and medium. Acceptor-dependent efflux was determined by subtracting the efflux in the absence of acceptor.

### ACAT activity

Two h after the addition of the SERMs or vehicle, an emulsion containing 12 μCi [9,10-^3^H]oleic acid (50 Ci/mmol, Hartmann Analytic, Braunschweig, Germany) and 1.5 mg/mL BSA was added to the media, and 24 h after the addition of the SERMs the cells were processed to determine the incorporation of [^3^H]oleate into cholesteryl esters as described^11^.

### Western blot analysis

Cells were lysed in 20 mM Tris-HCl buffer, pH 8.0, 120 mM KCl, 1 mM dithiothreitol, 1 mM Na_2_-EDTA, 2 mM EGTA, 0.1% Triton X-100, 0.5% Nonidet P40, and supplemented with a protease inhibitor cocktail. The cell lysate was sonicated and centrifuged at 4 °C to collect the supernatant. Liver samples were homogenized with a Polytron homogenizer in 10 mM HEPES, pH 7.6, at 4 °C, and the mixture was centrifuged at 600 × g for 10 min to collect the supernatant. For immunoblotting of ABCG5 and ABCG8, liver membranes were isolated by ultracentrifugation of the supernatant at 48,000 × g for 30 min at 4 °C in a Beckman TL-100 ultracentrifuge. The pellet was resuspended in 50 mM Tris-HCl buffer, pH 7.5, containing a protease inhibitor cocktail. Equal amounts of protein were subjected to western blot as described in Supplementary Methods.

### Real-time quantitative RT-PCR

Total RNA was extracted with the TriPure isolation reagent (Roche) and reverse transcribed with random hexamers using the PrimeScript RT reagent kit (Takara). Real-time PCR amplification was performed on a LightCycler 480 II using the SYBR Green I Master kit (Roche) according to the procedure previously described^69^ and using *RPLP0* (coding for ribosomal protein, large, P0) for human samples and *Cypb* (*Ppib*, coding for peptidylprolyl isomerase B) for mouse samples as invariant controls. Primer sequences for the different genes are shown in Supplementary Table S1.

### Fluorescence and optical microscopy

Cells were cultured on glass coverslips previously treated with poly-D-lysine. After the corresponding treatments, fixed cells were stained with filipin for free cholesterol as described previously^69^ and examined on an Olympus BX51 microscope. On other occasions, cells were costained with filipin and Bodipy for neutral lipids or were incubated in the presence of DiI-labeled AcLDL during the treatments and, finally, cells were mounted for microscopy and examined on a Nikon D-Eclipse C1 confocal microscope.

Liver pieces were fixed in 4% paraformaldehyde, cryoprotected and frozen before sectioning into 5 μm-thick sections on a cryostat. Liver sections were mounted on positively charged slides and stained with filipin. Other sections were stained with 0.3% Oil Red O in 60% isopropanol and then processed for hematoxylin counter staining.

### Macrophage RCT studies, *in vivo*

Studies of macrophage RCT were performed *in vivo* in 14-week-old male mice fed a western-type diet (Harlan Teklad) ad libitum for 4 weeks. The last 10 days on this diet, mice were given 10 mg/kg/day TAM, RAL or vehicle (1% carboxymethylcellulose) by oral gavage. Forty-eight hours before the end of the treatment, mice received an intraperitoneal injection of [^3^H]cholesterol-labeled peritoneal macrophages previously treated *ex vivo* with the same SERM or vehicle as the receptor mouse. These macrophages were prepared from male mice fed a western type diet for 4 weeks. Subsequently, macrophages were labeled with 5 μCi/mL [1,2-^3^H(N)]cholesterol, loaded with AcLDL-cholesterol (120 μg/mL) and, simultaneously, treated with TAM, RAL (10 μM) or vehicle in RPMI 1640 supplemented with 10% LPDS for 24 h. [^3^H]Cholesterol-labeled cells were then washed, equilibrated in RPMI 1640 containing 0.2% BSA for 4 h and injected intraperitoneally into recipient mice as previously reported^70^. Blood samples were taken at the indicated time points and ^3^H radioactivity was measured in serum. Unless otherwise specified, mice were killed 48 h after macrophage injection, liver was harvested and the total feces produced over the 48 h-RCT study was collected. ^3^H radioactivity in liver cholesterol and fecal cholesterol and bile acids were determined as described^70^.

### HDL kinetic studies

HDL (1.063–1.21 kg/L) was isolated by sequential ultracentrifugation of pooled plasma from male mice treated with a western-type diet and TAM, RAL or vehicle as described above and labeled with [1,2-^3^H(N)]cholesteryl oleate (Perkin-Elmer) as reported^56^,^57^. [1,2-^3^H(N)]cholesteryl oleate-labeled HDL were injected intravenously into mice (2 × 10^6^ dpm/mouse) that were being treated with the same SERM or vehicle as the HDL donor mouse (“autologous” HDL). The injection was given at day 8 of these treatments and blood samples were collected at the indicated times during the additional two days of treatment to measure ^3^H counts in serum. At the end of the experiment, ^3^H counts in liver cholesterol and fecal cholesterol and bile acids were also determined as described above.

### Lipid analyses

HDL-cholesterol levels were measured after precipitation of apoB-containing lipoproteins with phosphotungstic acid/MgCl_2_. To analyze HDL composition, these particles were isolated by ultracentrifugation (1.063–1.21 kg/L). Liver lipids were extracted from 100 mg of liver with isopropyl alcohol-hexane 2:3 (v/v) and, after the addition of Na_2_SO_4_, the hexane phase was isolated, dried with N_2_, reconstituted with 0.5% sodium cholate and sonicated for 10 min (50 Hz) before lipid measurements. Lipids were measured by enzymatic methods using a BM/Hitachi 917 autoanalyzer as described^57^. Protein concentration was measured using the BCA kit (Thermo Scientific). To determine the cellular content of total and free cholesterol, lipids were extracted, then subjected or not, respectively, to saponification and analyzed by gas chromatography-mass spectrometry as previously described^69^. Mass of cholesterol in esterified cholesterol was derived by difference. The cellular triacylglycerol mass was determined in lipid extracts by liquid chromatography with evaporative light scattering detection^71^.

### Statistical analysis

The effects of SERMs were analyzed by one-way ANOVA. For data assessed at various doses or time points, main and interactive effects were analyzed by two-way ANOVA. For *in vitro* experiments, one-way or two-way repeated measures ANOVA were used. Post hoc multiple comparisons were performed by Student–Newman–Keuls test. The analyses were performed using the SigmaStat 2.0 software. *P* ≤ 0.05 was defined as statistically significant.

## Additional Information

**How to cite this article**: Fernández-Suárez, M. E. *et al*. Clinically used selective estrogen receptor modulators affect different steps of macrophage-specific reverse cholesterol transport. *Sci. Rep.* **6**, 32105; doi: 10.1038/srep32105 (2016).

## Supplementary Material

Supplementary Information
