# Supplementary material for: Clinically used selective estrogen receptor modulators affect different steps of macrophage-specific reverse cholesterol transport
Source: Sci Rep. 2016 Sep 7;6:32105. doi: 10.1038/srep32105 (PMC5013287; doi:10.1038/srep32105)
Supplement: Supplementary Information [file srep32105-s1.pdf]

Clinically used selective estrogen receptor modulators affect different steps of macrophage-specific reverse cholesterol transport

María E. Fernández-Suárez, Joan C. Escolà-Gil, Oscar Pastor, Alberto Dávalos, Francisco Blanco-Vaca, Miguel A. Lasunción, Javier Martínez-Botas & Diego Gómez-Coronado

Supplementary Methods

Human monocyte-derived macrophages After Lymphoprep (Nycomed Pharma AS) density gradient centrifugation of buffy coats[1], the mononuclear cells were resuspended in RPMI 1640 supplemented with 1% human male AB+ serum (Sigma-Aldrich), plated on human serum-treated flasks and incubated for 2 h. Nonadherent cells were removed by washing with PBS and adherent cells were then detached by incubation in PBS containing 10% human serum and 0.02% Na_2-EDTA for 40 min at 8ºC. Subsequently, these cells were extensively washed with PBS and resuspended in RPMI 1640 supplemented with 10% human male AB+ serum, 2 mM glutamine, 100 U/mL penicillin, 100 U/mL streptomycin and 10 µg/mL gentamicin. They were plated at a density of 10^6 cells/mL and incubated at 37°C in a humidified atmosphere of 5% CO_2 for 7 days to allow monocytes to differentiate into macrophages.

Mouse peritoneal macrophages Peritoneal macrophages were obtained by lavage with PBS 3 days after intraperitoneal injection with 1 mL of 3% thioglycolate. The cells were plated at a density of 5 x10^5 cells/mL and allowed to adhere by incubation with DMEM supplemented with 1% FBS for 2 h. After removal of nonadherent cells by washing with PBS, the cells were incubated in DMEM supplemented with 10% FBS at 37°C in a humidified atmosphere of 5% CO_2 for 3 days before using them for the experiments.

Western blot analysis Equal amounts of protein were subjected to SDS/10% PAGE, transferred to a nitrocellulose filter and probed with antibodies directed against ABCA1, ABCG1, calnexin (Abcam), ABCG8, SR-BI, CD36, NPC1 (Novus Biologicals), β-actin, GAPDH (Santa Cruz Biotechnology) or ABCG5 (a generous gift from Dr. Albert K. Groen, Department of Pediatrics, University Medical Center Groningen, University of Groningen, Groningen, The Netherlands). Bound antibodies were visualized using secondary antibodies conjugated to IRDye 800CW or IRDye 680LT and the Odyssey Infrared Imaging System (LI-COR). The density of the bands was quantified by using the Quantity One 4.5.2 program (Bio-Rad).

Supplementary Table S1. Primer sequences for real-time quantitative RT-PCR.

Gene	NCBI Reference Sequence	Primer	Sequence
Homo sapiens			
ABCA1	NM_005502	ABCA1 Fw	5'-GCACTGAGGAAGATGCTGAAA-3'
		ABCA1 Rev	5'-AGTTCCTGGAAGGTCTTGTTCA-3'
ABCG1	NM_004915	ABCG1 Fw 1.1	5'-TTCTGACATTTCCCCTGGAG-3'
		ABCG1 Rev 1.1	5'-CAGTAGGCCACTGGGAACAT-3'
RPLP0	NM_001002<br>NM_053275	RPLP0 Fw	5'-CCTCATATCCGGGGGAATGTG-3'
		RPLP0 Rev	5'-GCAGCAGCTGGCACCTTATTG-3'
Mus musculus			
Abca1	NM_013454	Abca1 Fw 1.1	5'-GTTACGGCAGATCAAGCATCC-3'
		Abca1 Rev 1.1	5'-TGGAAGGGACAAATTGTGCTG-3'
Abcg1	NM_009593	Abcg1 Fw 1.1	5'-GGGGAAAGGTCTCCAATCTC-3'
		Abcg1 Rev 1.1	5'-TGTTCTGATCCCCGTACTCC-3'
Abcg5	NM_031884	Abcg5 Fw 1.1	5'-CGCGAGACGTTGCGATACA-3'
		Abcg5 Rev 1.1	5'-CTGCCAATCATTTGGTCCGC-3'
Abcg8	NM_026180	Abcg8 Fw 1.1	5'-CTGTGGAATGGGACTGTACTTC-3'
		Abcg8 Rev 1.1	5'-GTTGGACTGACCACTGTAGGT-3'
Cyp7a1	NM_007824	mCyp7a1 Fw 2.1	5'-CTGTCATACCACAAAGTCTTATGTCA-3'
		mCyp7a1 Rev 2.1	5'-ATGCTTCTGTGTCCAAATGCC-3'
Cypb	NM_011149	mCypb Fw 1.1	5'-TGGAGAGCACCAAGACAGACA-3'
		mCypb Rev 1.1	5'-TGCCGGAGTCGACAATGAT-3'
Scarb1	NM_016741<br>NM_001205082<br>NM_001205083	SCARB1 Fw 1.1	5'-TGTACTGCCTAACATCTTGGTCC-3'
		SCARB1 Rev 1.1	5'-ACTGTGCGGTTCATAAAAGCA-3'

Reference

1. Contreras J.A., Lasuncion M.A. Essential differences in cholesteryl ester metabolism between human monocyte-derived and J774 macrophages. Evidence against the presence of hormone-sensitive lipase in human macrophages. *Arterioscler. Thromb.* **14,** 443-452 (1994).

Supplementary Figures

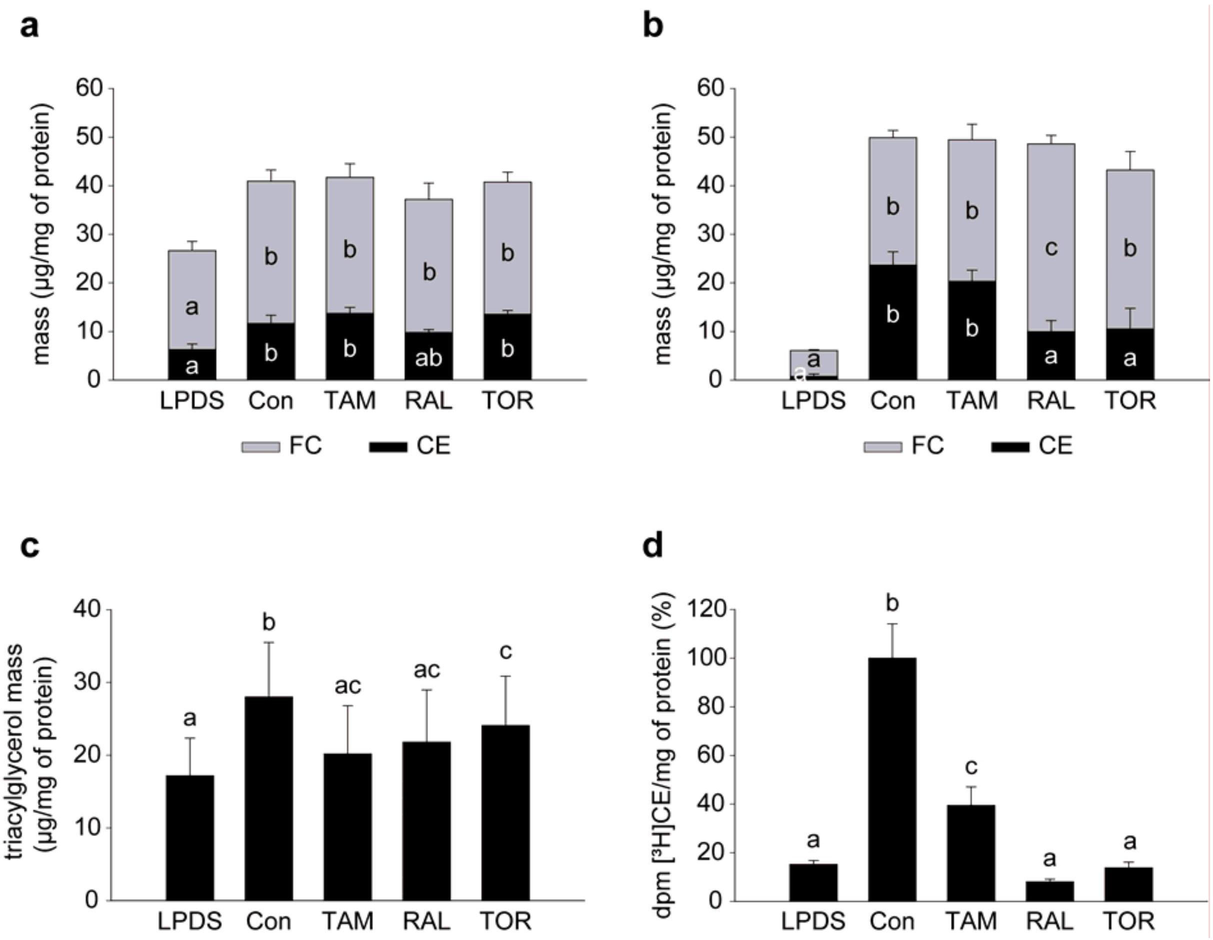

Supplementary Figure S1. Effects of SERMs on cholesterol and triacylglycerol contents and ACAT activity of THP-1 macrophages exposed to AcLDL. THP-1 macrophages were treated without (LPDS) or with AcLDL plus vehicle (Con) or tamoxifen (TAM), raloxifene (RAL) or toremifene (TOR) (10 µM) for 24 h (**a**, **c** and **d**) or 48 h (**b**). At the end of the treatments, the mass of total, free (FC) and esterified cholesterol (CE) (**a** and **b**) or triacylglycerols (**c**) was determined. ACAT activity (**d**) was measured after the addition of [^{3}H]oleate to the medium as indicated in Materials and Methods; dpm of [^{3}H]cholesteryl esters (CE)/mg of cell protein was calculated and expressed as % of the value of the control condition (Con) of the same experiment. Data are mean±SEM of 3 (**a**) or 4 (**b**, **c** and **d**) independent experiments. Bars or sections not sharing any letter are statistically different (*P*<0.05).

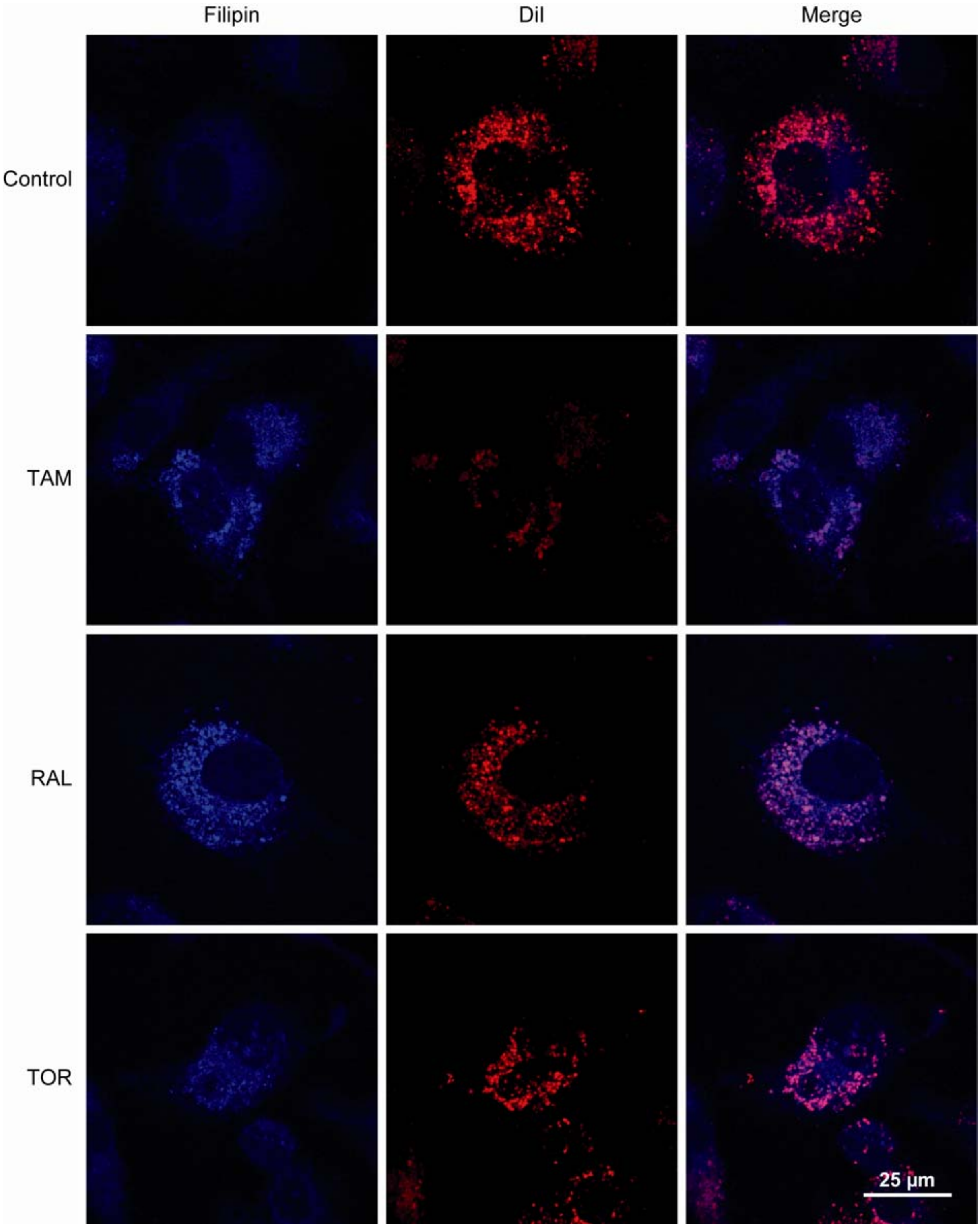

Supplementary Figure S2. Effect of SERMs on the colocalization of free cholesterol with DiI-AcLDL in THP-1 macrophages. Cells were treated with DiI-AcLDL and vehicle (control) or tamoxifen (TAM), raloxifene (RAL) or toremifene (TOR) (10 µM). Then cells were stained with filipin for free cholesterol and examined for filipin and DiI fluorescence. Photographs are representative examples from three independent experiments.

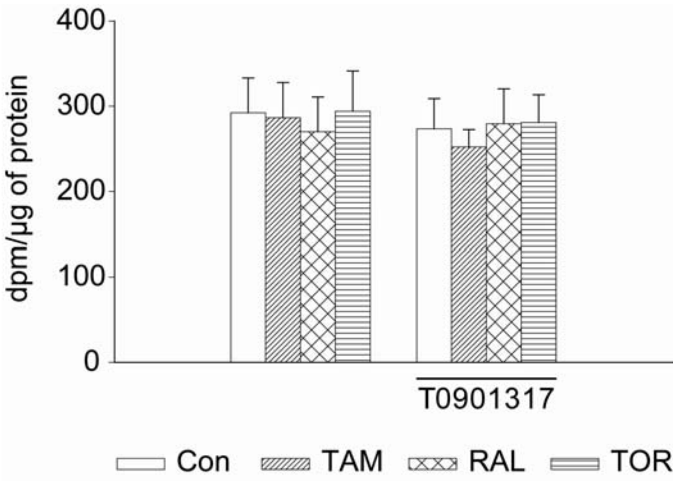

Supplementary Figure S3. Effect of SERMs on THP-1 macrophage labeling with [^{3}H]cholesterol-AcLDL. Cells were treated with [^{3}H]cholesterol-AcLDL and vehicle (Con) or tamoxifen (TAM), raloxifene (RAL) or toremifene (TOR) (10 μM) and in the absence or presence of T0901317 (1 μM). Then cells were washed and [^{3}H]cholesterol levels were measured. Data are mean±SEM of 3 independent experiments. dpm indicates disintegrations per minute.

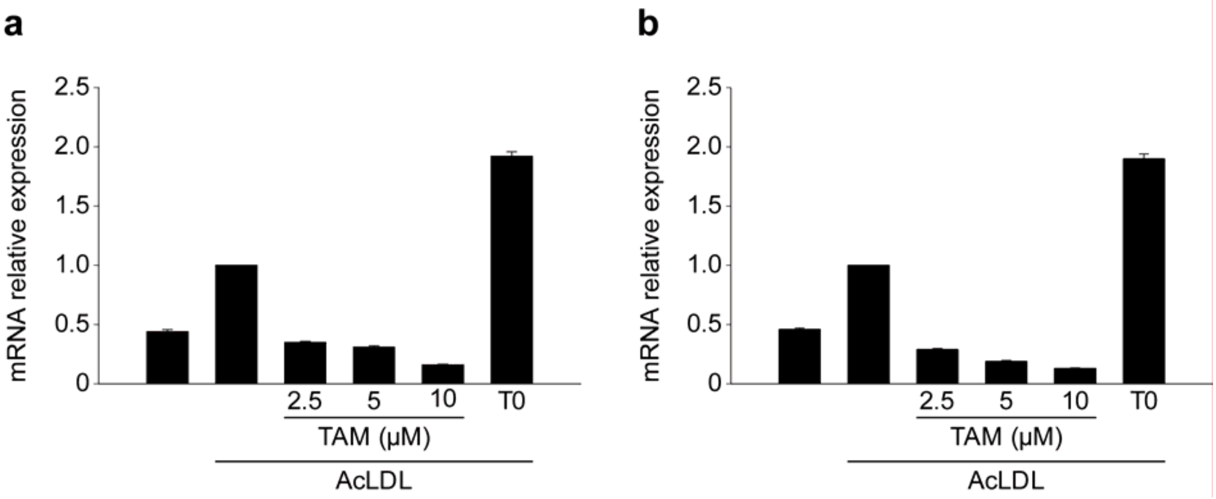

Supplementary Figure S4. Dose-dependent effect of tamoxifen on ABCA1 and ABCG1 gene expression in THP-1 macrophages. Cells were incubated in the absence or presence of AcLDL and the indicated concentrations of tamoxifen (TAM) or T0901317 (T0, 1 μM), and the gene expression of ABCA1 (**a**) and ABCG1 (**b**) were measured by real-time RT-PCR. Data are mean±SEM of a triplicate determination from a representative experiment of the two that were carried out and are expressed as the relative amount of mRNA compared to the amount in the presence of AcLDL alone.

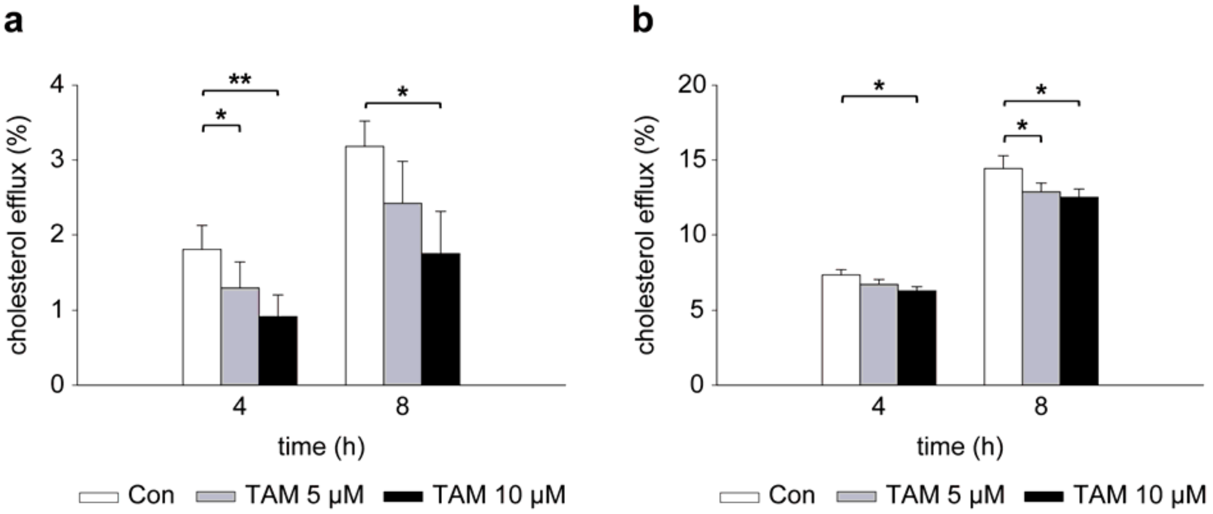

Supplementary Figure S5. Effect of SERMs on cholesterol efflux from THP-1 macrophages labeled with [^{3}H]cholesterol added in ethanol. After labelling with [^{3}H]cholesterol, cells were treated with AcLDL and vehicle (Con) or the indicated concentrations of tamoxifen (TAM). Then cholesterol efflux was measured in the presence of apoA-I (**a**) or HDL (**b**) at 4 or 8 h. Data are mean±SEM from 6 independent experiments. * $P<0.05$, ** $P<0.01$.

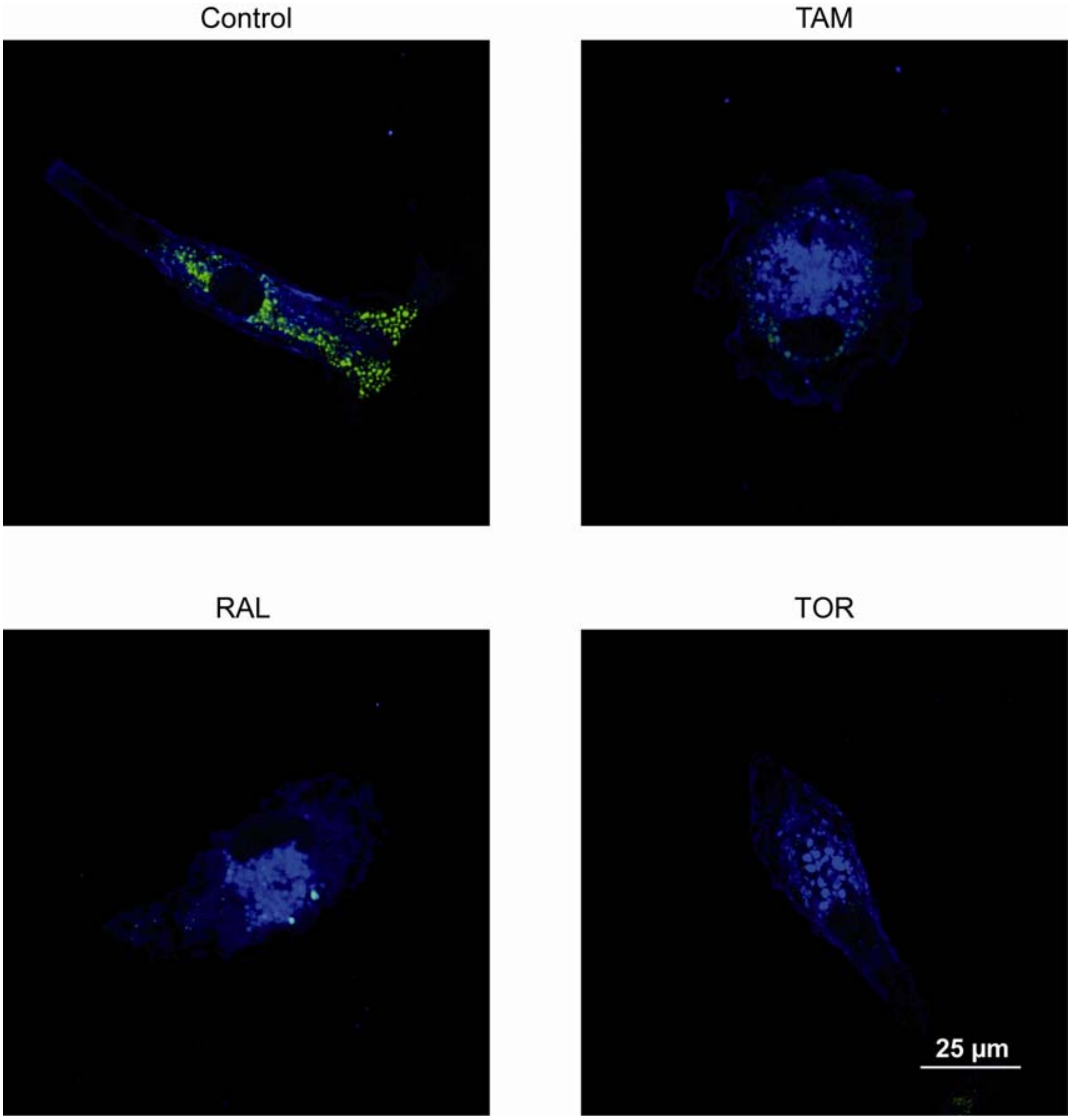

Supplementary Figure S6. Effect of SERMs on free cholesterol and nonpolar lipid distribution in human monocyte-derived macrophages. Cells were treated with AcLDL and vehicle (Control) or tamoxifen (TAM), raloxifene (RAL) or toremifene (TOR) (10 µM). Then cells were stained with filipin for free cholesterol (blue) and Bodipy for nonpolar lipids (green) and analyzed by confocal microscopy. Photographs are representative examples from three independent experiments.

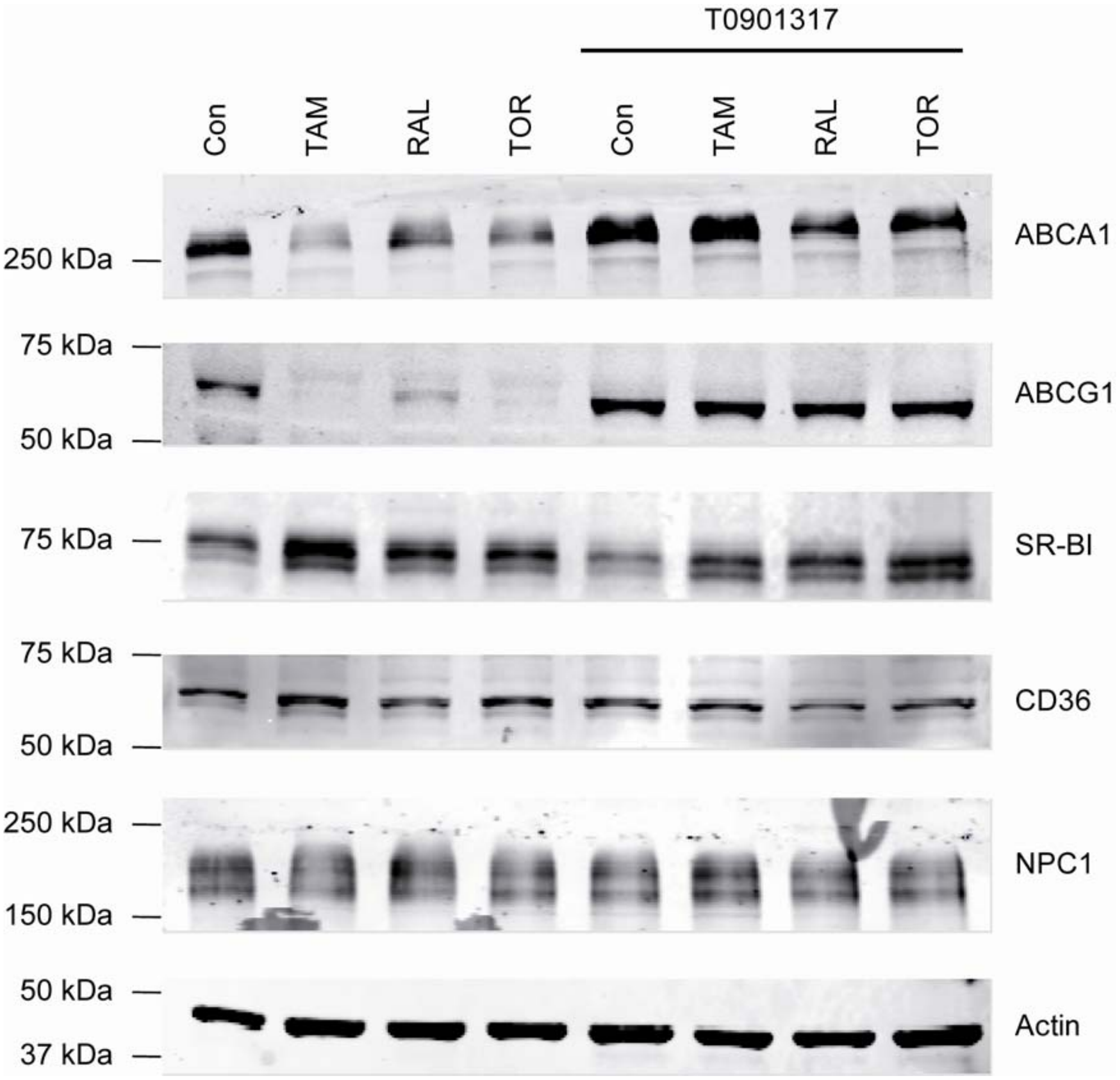

Supplementary Figure S7. Effect of SERMs on the protein expression of cholesterol transporters in human monocyte-derived macrophages. Cells were treated with AcLDL and vehicle (Con) or tamoxifen (TAM), raloxifene (RAL) or toremifene (TOR) (10 μM) and in the absence or presence of T0901317 (1 μM). Results are representative examples from three independent experiments.

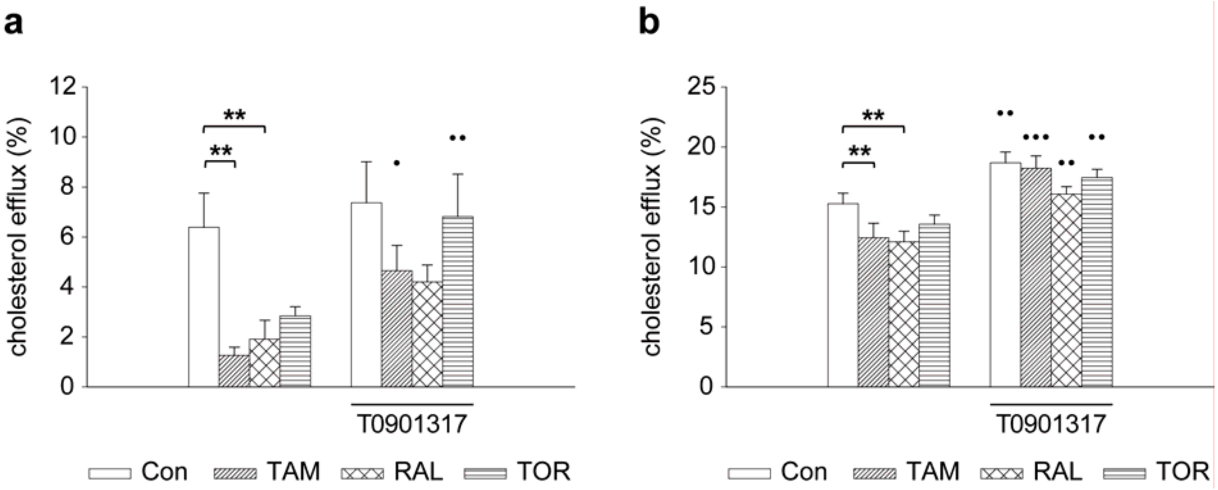

Supplementary Figure S8. Effect of SERMs on AcLDL-derived cholesterol efflux from human monocyte-derived macrophages. Cells were treated with [^{3}H]cholesterol-AcLDL and vehicle (Con) or tamoxifen (TAM), raloxifene (RAL) or toremifene (TOR) (10 µM) and in the absence or the presence of T0901317 (1 µM). Then, apoA-I (**a**) or HDL (**b**) were added to the medium and cholesterol efflux was measured at 24 h. Data are mean±SEM of macrophages from 5 subjects. ** *P*<0.01, *** *P*<0.001; • *P*<0.05, •• *P*<0.01, ••• *P*<0.001 between conditions only differing in the presence of T0901317.

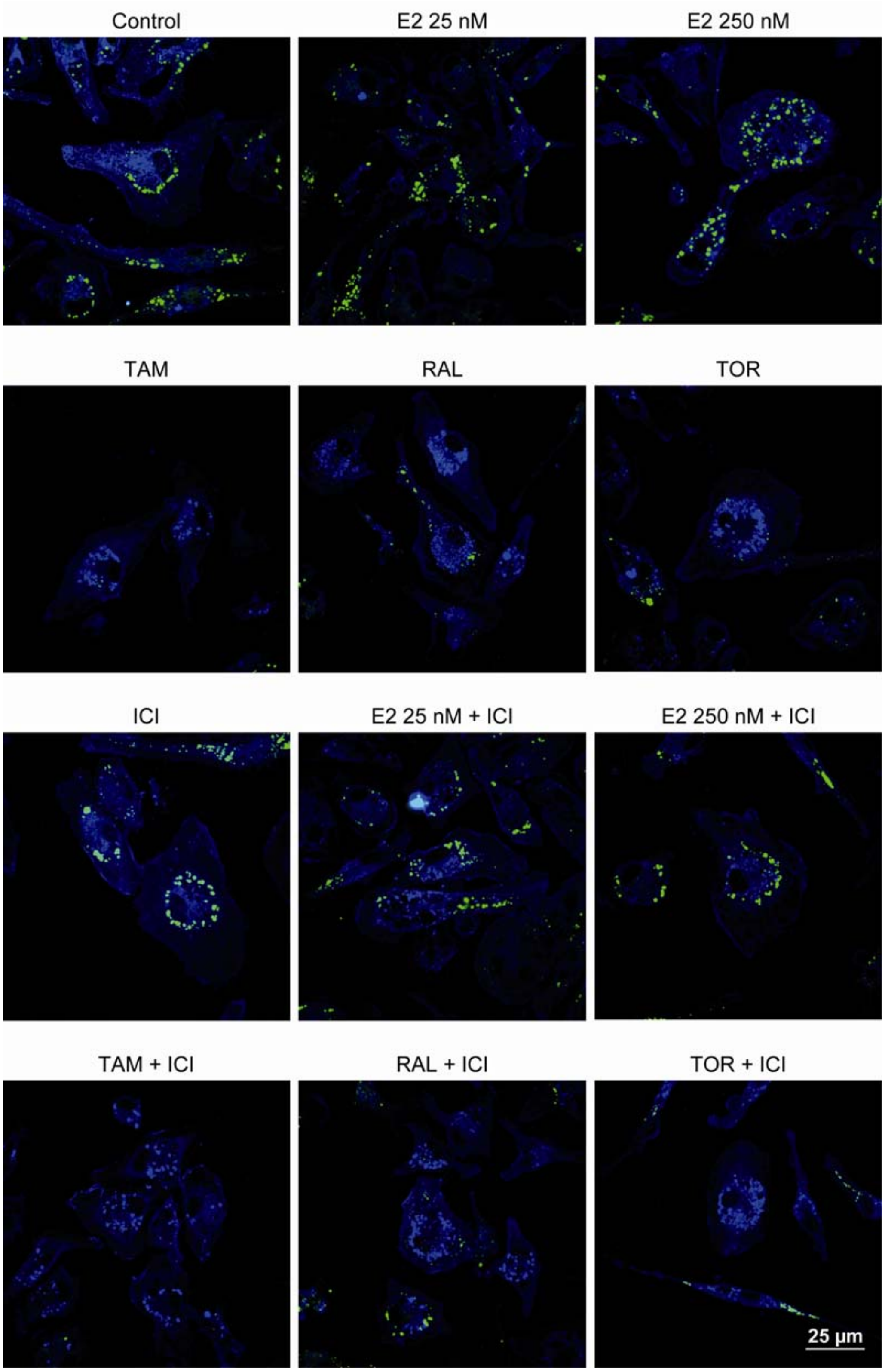

Supplementary Figure S9. Effect of SERMs, ICI 182,780 and 17β-estradiol on free cholesterol and nonpolar lipid distribution in mouse peritoneal macrophages. Cells were treated with AcLDL and vehicle (Control) or tamoxifen (TAM), raloxifene (RAL) or toremifene (TOR) (10 µM), or the indicated concentrations of 17β-estradiol (E2) and in the absence or the presence of ICI 182,780 (ICI, 1 µM). Then cells were stained with filipin for free cholesterol (blue) and Bodipy for nonpolar lipids (green) and analyzed by confocal microscopy. Photographs are representative examples from three independent experiments.

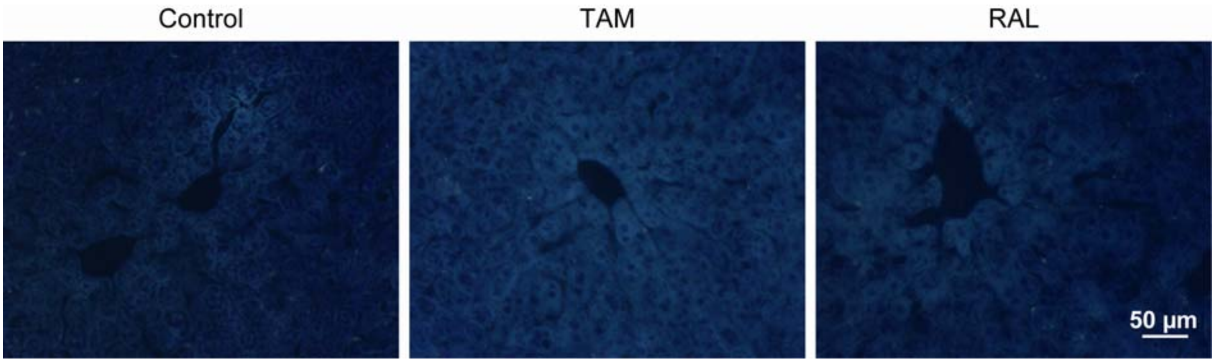

Supplementary Figure S10. Effect of tamoxifen and raloxifene on free cholesterol distribution in liver sections. Mice were fed a western-type diet for 4 weeks and were treated with tamoxifen (TAM), raloxifene (RAL) or vehicle (Control) for the last 10 days. Liver sections were stained with filipin for free cholesterol and analyzed by confocal microscopy. Photographs are representative examples from three mice per group.
